# ^18^F-Labeled Silicon-Based Fluoride Acceptors: Potential Opportunities for Novel Positron Emitting Radiopharmaceuticals

**DOI:** 10.1155/2014/454503

**Published:** 2014-07-24

**Authors:** Vadim Bernard-Gauthier, Carmen Wängler, Esther Schirrmacher, Alexey Kostikov, Klaus Jurkschat, Bjoern Wängler, Ralf Schirrmacher

**Affiliations:** ^1^Division of Experimental Medicine, Department of Medicine, McGill University, 1110 Pine Avenue West, Montreal, QC, Canada H3A 1A3; ^2^Biomedical Chemistry, Department of Clinical Radiology and Nuclear Medicine, Medical Faculty Mannheim of Heidelberg University, 68167 Mannheim, Germany; ^3^McConnell Brain Imaging Centre, Montreal Neurological Institute, McGill University, 3801 University Street, Montreal, QC, Canada H3A 2B4; ^4^Department of Inorganic Chemistry II, Faculty of Chemistry, TU Dortmund, Otto-Hahn-Straße 6, 44221 Dortmund, Germany; ^5^Molecular Imaging and Radiochemistry, Department of Clinical Radiology and Nuclear Medicine, Medical Faculty Mannheim of Heidelberg University, 68167 Mannheim, Germany; ^6^Department of Oncology, University of Alberta, 11560 University Avenue, Edmonton, AB, Canada T6G 1Z2

## Abstract

*Background.* Over the recent years, radiopharmaceutical chemistry has experienced a wide variety of innovative pushes towards finding both novel and unconventional radiochemical methods to introduce fluorine-18 into radiotracers for positron emission tomography (PET). These “nonclassical” labeling methodologies based on silicon-, boron-, and aluminium-^18^F chemistry deviate from commonplace bonding of an [^18^F]fluorine atom (^18^F) to either an aliphatic or aromatic carbon atom. One method in particular, the silicon-fluoride-acceptor isotopic exchange (SiFA-IE) approach, invalidates a dogma in radiochemistry that has been widely accepted for many years: the inability to obtain radiopharmaceuticals of high specific activity (SA) via simple IE. *Methodology.* The most advantageous feature of IE labeling in general is that labeling precursor and labeled radiotracer are chemically identical, eliminating the need to separate the radiotracer from its precursor. SiFA-IE chemistry proceeds in dipolar aprotic solvents at room temperature and below, entirely avoiding the formation of radioactive side products during the IE. *Scope of Review.* A great plethora of different SiFA species have been reported in the literature ranging from small prosthetic groups and other compounds of low molecular weight to labeled peptides and most recently affibody molecules. *Conclusions.* The literature over the last years (from 2006 to 2014) shows unambiguously that SiFA-IE and other silicon-based fluoride acceptor strategies relying on ^18^F^−^ leaving group substitutions have the potential to become a valuable addition to radiochemistry.

## 1. Introduction

Radiopharmaceutical chemistry, besides the medicinal rationale, is undoubtedly the driving force behind tracer development for in vivo molecular imaging. Devising new radiochemical methodologies to introduce radioisotopes into organic molecules of various molecular weights and chemical nature has been a continuing strife throughout the history of radioactive probe development. In principle, almost any organic compound can be radioactively labeled depending on the nuclide, the acceptable level of derivatization which is necessary particularly in radiometal labeling, and of course the position of the label itself. With the contingent of existing labeling methods, it is possible to label nearly all compounds in sufficient radiochemical yields (RCYs); however, sometimes the required great technical effort can prevent clinical routine production. Currently, only radiochemistries based on coordinating radiometals such as technetium-99m (^99m^Tc), which accounts for the majority of all radiopharmaceuticals produced for single-photon emission computed tomography (SPECT), as well as indium-111 (^111^In, for SPECT), gallium-68 (^68^Ga), and copper-64 (^64^Cu) both for positron emission tomography (PET) proceeds in a kit-like manner [1–4]. In particular, ^99m^Tc radiochemistry evolved over decades into fully GMP compliant (Good Manufacturing Practice) labeling kits where a simple addition of the radionuclide in the chemical form of its pertechnetate (^99m^TcO_4_^−^) followed by very few simple steps yields the tracer. For other radiometals, final HPLC purification is sometimes inevitable and the operators in the laboratory have to possess a certain degree of technical proficiency and equipment in order to deliver an injectable solution that complies with GMP regulations.

Additional obstacles exist for radiolabeling with the most extensively used PET isotope ^18^F. The interest towards the development of ^18^F-radiopharmaceuticals ensues essentially from the low positron energy (635 KeV) and the most suitable half-life (109.7 min) of this radioisotope. As a consequence, ^18^F is ideal for numerous PET imaging applications involving tracers of low molecular weight as well as various biomolecules with a suitable kinetic profile. In particular, the successful and widespread use of [^18^F]2-fluoro-2-deoxy-D-glucose ([^18^F]FDG) has ignited the interest in new ^18^F-tracers but despite its favorable nuclear properties, ^18^F-radiochemistry remains often associated with relatively cumbersome and lengthy labeling procedures. Indeed, ^18^F-labeling normally involves relatively large precursor quantities and often requires high reaction temperatures as well as the presence of activating reagents (e.g., strong bases plus cryptands) leading to unwanted radioactive and chemical side products, which need to be thoroughly separated from the desired ^18^F-labeled tracer. Consequently, there are only few examples published in the literature where the radiochemical labeling procedure does not require a final HPLC purification. This is problematic due to the need for fully GMP compliant synthesis modules, which led manufacturers to search for solid phase based purifications to circumvent HPLC procedures [5–7]. Moreover, the classical use of harsh reaction conditions precludes a direct ^18^F-radiolabeling of complex biomolecules not able to withstand those reaction conditions. In such cases, the use of ^18^F-carbon-based prosthetic groups is often necessary, imposing further equipment challenges in addition to the time-consuming aspects.

**Scheme 1:**
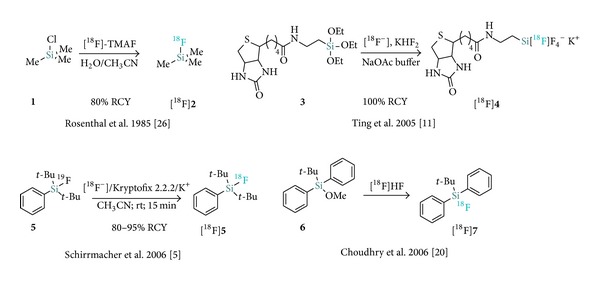
Early developments of silicon-[^18^F]fluorine-based compounds.

The recent development of comparatively simple, efficient, and innovative labeling approaches based on silicon-^18^F [5, 8–10] and boron-^18^F [11–14] bond formation as well as aluminium-^18^F [14–19] chelation scaffolds each address in part some of the major drawbacks associated with conventional nucleophilic ^18^F-labeling on a carbon atom. Particularly, silicon-^18^F labeling methods have been increasingly exploited in recent years due to their inherent simplicity and efficiency compared to conventional labeling strategies. The organosilicon-based fluoride acceptor (SiFA) ^18^F-labeling strategy was initially coined in reference to the isotopic exchange (IE) approach introduced by Schirrmacher et al. [5] (Scheme 1). A more inclusive definition of SiFAs also comprises the alkoxysilane leaving group approach introduced by Choudhry et al. [20] which was expanded to hydrosilanes and silanols by the group of Ametamey [21]. The current review will detail and discuss the technical developments and applications which have led to the current status of [^18^F]-SiFA radiochemistry as a simplified approach towards new radiopharmaceuticals for PET imaging.

## 2. SiFA Labeling Chemistry

Formation of Si–F bonds is driven by the strong affinity between silicon and fluorine as exemplified by the high corresponding bond energy (565 kJ mol^−1^ for Si–F versus 485 kJ mol^−1^ for C–F and 318 kJ mol^−1^ for Si–C). Simple organofluorosilanes display poor kinetic stability and may be cleaved under mild conditions in the presence of fluoride or other silophiles due to the high polarization of Si–F bonds which is also true for Si–O bonds. Tetravalent silicon readily reacts with Lewis bases to form hypervalent species (both 5- and 6-coordinate) as a consequence of vacant low energy d-orbitals. Moreover, the greater covalent radius of silicon versus carbon contributes to the enhanced propensity of organosilanes to undergo nucleophilic substitutions at the silicon atom compared to their carbon-centered counterparts. Those characteristics build the foundation of various nonradioactive organosilicon chemistries and are also central to the development of [^18^F]organofluorosilanes for PET imaging.

The synthesis of ^18^F-labeled silicon tetrafluoride (Si[^18^F]F_4_) via metallic hexafluoridosilicate formation from metallic fluorides and SiF_4_ has been known for more than half a century in radiochemistry [22–24]. [^18^F]Fluorotrimethylsilane ([^18^F]**2**) was also reported as a hypothetical intermediate from hexamethylsiloxane reaction with [^18^F]HF as early as 1978 [25]. The first in vivo evaluation of silicon-^18^F building blocks was introduced by Rosenthal et al. with the radiosynthesis of the volatile species [^18^F]**2** from chlorotrimethylsilane (**1**; Scheme 1) [26]. The labeling proceeded efficiently delivering [^18^F]**2** using no-carrier-added (nca) tetramethyl-ammonium-[^18^F]fluoride ([^18^F]TMAF, 80% radiochemical yield (RCY) decay-corrected); however, upon inhalation by rats extensive bone uptake was observed as a result of defluorination (anionic ^18^F− is rapidly taken up by the bone apatite). This result paralleled the observed poor hydrolytic in vitro profile of [^18^F]**2** which led the authors at the time to suggest that bulkier groups at the silicon atom may be necessary in order to generate hydrolytically stable ^18^F-silicon building blocks. This original contribution was followed by the development of variations of [^18^F]fluorotrimethylsilane-based release of dry nca ^18^F^−^ for the use in nucleophilic radiofluorination [27, 28].

In a more recent study, the group of Perrin provided an innovative approach towards ^18^F-silicon building blocks, synthesizing the biotin-linked alkyl tetrafluorosilicate [^18^F]**4** via near-quantitative carrier added radiofluorination (from KHF_2_) [11]. A typical reaction procedure involved reacting alkyl triethoxysilane** 3** with a preformed mixture of 440 *μ*Ci of ^18^F^−^/H_2_O from target water ([^18^O]H_2_O) along with 130 mM KHF_2_ (4.4 equiv.) in 200 mM NaOAc (pH 4.5). This important development also constituted the first efficient ^18^F^−^ aqueous labeling and provided, despite hydrolytic stability concerns, the groundwork for ^18^F-silicon radiochemistry developments.

In 2006, Schirrmacher et al. convincingly demonstrated that [^18^F]SiFA building blocks could be generated in high RCYs and specific activity (SA) by means of a IE from the corresponding- and chemically identical-^19^F-precusors [5]. Conversion of [^19^F]-*t*Bu_2_PhSiF (**5**) to the radiolabeled [^18^F]-*t*Bu_2_PhSiF ([^18^F]**5**) proceeded in 80–95% RCYs in the presence [^18^F]^−^/Kryptofix 2.2.2/K^+^ in acetonitrile (100 *μ*L) with minimal precursor quantity (1 *μ*g). The prototypical di-*tert*-butylphenyl-bearing SiFA [^18^F]**5** was obtained in SAs of 2.7–27 Ci *μ*mol^−1^ and the methodology was also applied to direct, unprotected labeling of SiFA-aminooxy-derivatized Tyr^3^-octreotate at room temperature (see Section 4). This work validated that IE at the silicon atom (SiFA-IE) constitutes an effective and mild methodology towards new ^18^F-labeled compounds. The authors also reported an early stability study of a series of labeled SiFA derivatives (*vide infra*). This result was reported almost simultaneously with the important contribution of Choudhry et al. establishing a silicon-leaving group approach to the radiosynthesis of [^18^F]SiFA starting from an alkoxy-substituted acceptor precursor [20]. The reaction proceeded directly from aqueous ^18^F^−^ and allowed for the efficient conversion of* tert*-butyldiphenylmethoxysilane (**6**) to [^18^F]*tert*-butyldiphenylfluorosilane ([^18^F]**7**) at room temperature in 5 minutes.

**Figure 1:**
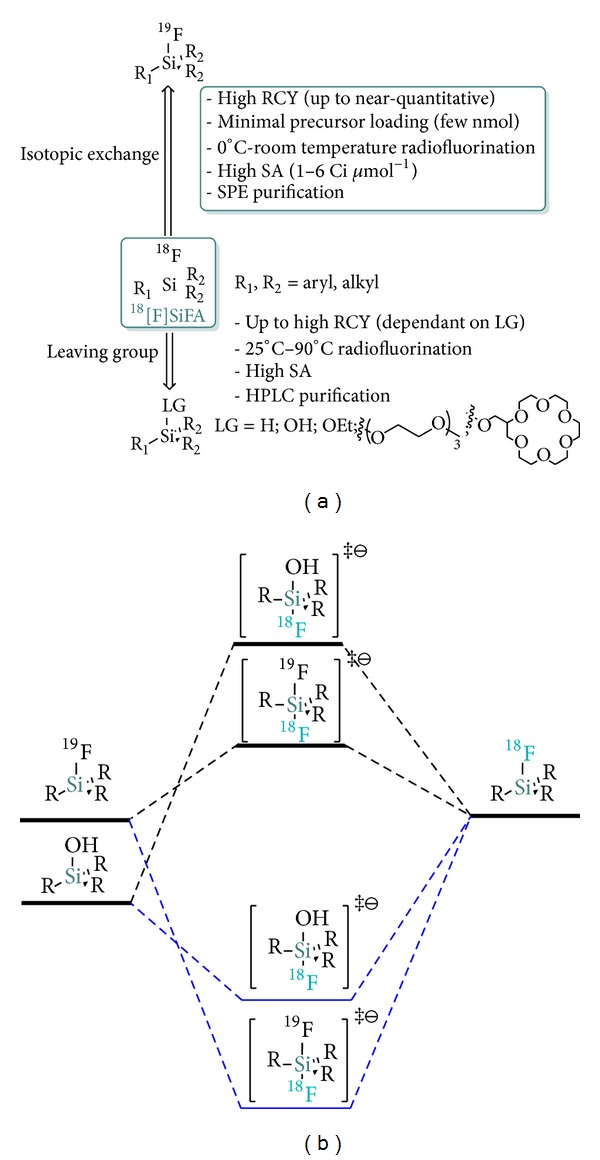
(a) Approaches towards [^18^F]SiFA compounds for PET; [^18^F]SiFA can be obtained by either isotopic exchange or leaving group substitution from the suitable organosilane precursors. (b) Comparison of simplified reaction coordinates for IE and leaving group radiofluorination (from hydroxysilane in the absence of acid catalyst). Simple hypothetical siliconates intermediates are depicted. (Gas phase = dash blue lines, MeCN = dash black lines.)

The leaving group methodology currently constitutes one of the two extensively exploited strategies towards [^18^F]SiFAs—the other one being the SiFA-IE. Both approaches were shown to deliver [^18^F]SiFA in high RCYs and SAs (Figure 1(a)). Yet, important distinctions exist between the two techniques, one of which resides in the fact that the IE typically proceeds at room temperature or below while the Si-leaving group approach, like aliphatic and aromatic ^18^F-carbon radiochemistry in general, necessitates elevated temperatures which may be detrimental when direct labeling of biomolecules is considered.

**Figure 2:**
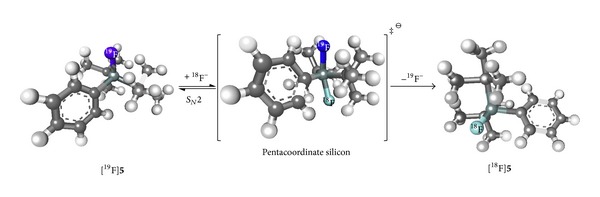
Formation of a trigonal bipyramid siliconate anion intermediate leading to formation of [^18^F]**5** from [^19^F]**5** (gray = carbon; dark green = silicon; blue = fluorine-19; cyan = fluorine-18; white = hydrogen).

The efficiency of the IE, even at low temperatures, can be attributed to the low energy barrier for the ^19^F^−^ isoenergetic replacement with ^18^F^−^ in acetonitrile via the formation of a trigonal bipyramidal siliconate anion intermediate (Δ*G*_IE_ ≈ 0; negligible isotopic effect; Figure 2). Indeed, DFT calculations in condensed phase (acetonitrile) on model SiFAs of the type R_3_SiF_2_^−^ indicated that Δ*G* ^‡^ values associated with the formation of siliconate intermediates from those precursors range from 5 to 10 kcal mol^−1^ (Figure 1(b), upper path) [29]. On the other hand, in the gas phase, values of Δ*G* ^‡^ of −50 to −40 kcal mol^−1^ were calculated in agreement with the expected formation of thermodynamically stable organofluorosiliconates (Figure 1(b), lower path) [30, 31]. Those energetic differences ensue from the diminished Lewis basicity of the fluoride anion in acetonitrile compared to that in the gas phase, suggesting that in the former case equilibrium is rapidly reached leading to the fast and near-irreversible formation of [^18^F]SiFA species as a consequence of stoichiometric leverage. Kostikov et al. also experimentally determined a characteristically low activation energy (*E*_*a*_ = 15.7 kcal mol^−1^) and exceptionally low preexponential factor (*A* = 7.9 × 10^13^ M^−1^ s^−1^) for the SiFA-IE from the corresponding Arrhenius plot [32]. These results are in contrast with the values gained from a comparable carbon-^18^F bond formation reaction, namely, the ^18^F-fluorination of ethyleneglycol-di-*p*-tosylate (*E*_*a*_ = 17.0 kcal mol^−1^ and *A* = 2.9 × 10^9^ M^−1^ s^−1^), and support the experimental observation that SiFA-IE proceeds quasi-quantitatively in many instances even at low temperatures [32]. In contrast, ^18^F-radiofluorination of more stable silanol precursors [33] (or other leaving group bearing silanes) should be endergonic (Δ*G* > 0) and associated with less stable hydrosiliconate intermediates in both gaseous and condense phases as expected from bond dissociation energies (BDEs).

An additional important distinction between IE and the leaving group method relates to purification techniques. Since the IE involves chemically identical entities and proceeds under mild conditions that do not lead to side products, HPLC purification can often be avoided and purification can be limited to solid phase cartridge extraction (SPE). This approach is feasible irrespective of the nature of the tracer (e.g., small fragments or biomolecules). In contrast, HPLC purification constitutes a prerequisite of the leaving group approach as chemically distinct precursors and ^18^F-radiolabeled products have to be carefully separated. Nevertheless, this method has been thoroughly developed and adapted frequently by the radiochemistry community. Since the initial contribution of Choudhry et al., the group of Ametamey and coworkers has further extended the silicon-leaving group approach methodology to hydride, hydroxy, and alkoxy leaving groups.

**Scheme 2:**
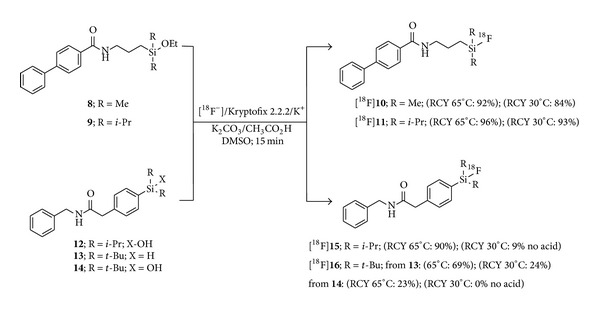
Synthesis of ^18^F-labeled silicon-containing model compounds with alkyl and aryl linkers by Mu et al.

Mu et al. exemplified this method with the radiosynthesis of a series of fluorosilanes bearing alkyl ([^18^F]**10**, [^18^F]**11**) or aryl ([^18^F]**15**, [^18^F]**16**) Si-linked fragments containing various R groups (Scheme 2) [21]. Few compounds such as the dimethyl- (**8**) and diisopropylethoxysilane (**9**) reacted readily at 30°C whereas most substrates necessitated elevated temperatures (65°C–90°C) in order to react with the ^18^F^−^. Compounds [^18^F]**15** and [^18^F]**16** were obtained in moderate to high RCYs from the corresponding silanol and silanes (SA of [^18^F]**16** = 1.73 Ci *μ*mol^−1^). As expected, adding acetic acid significantly influenced incorporation yields in the presence of* O*-bearing leaving groups but did not modify hydride rate departure from precursor** 13**.

**Scheme 3:**
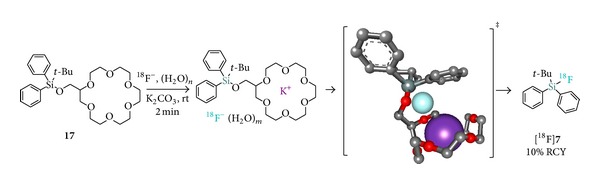
Postulated mechanism for rate enhancement in silicon fluorination using a crown ether leaving group by Al-huniti et al. conditions (gray = carbon; red = oxygen; dark green = silicon; cyan = fluoride; purple = potassium; hydrogen omitted for simplicity).

In a recent study, the leaving group SiFA methodology was combined with the nucleophile assisting leaving group (NALG) strategy to generate Si-appended potassium-chelating SiFA-based leaving groups [34, 35]. In the absence of added Kryptofix 2.2.2, the facilitation of ^18^F-fluorination in the presence of cyclic crown ethers such as in** 17** compared to acyclic polyethers or alkoxide leaving groups was clearly established. Unfortunately, the RCYs were undermined by the limited solubility (1–5%) of nca K^18^F in the reaction media. This issue was partially addressed by water addition (up to 0.5% v/v) leading to an increased K^18^F solubility (31%), but further addition subsequently diminished the observed RCYs. Consequently, upon optimized conditions, [^18^F]**7** was obtained in overall 10% RCY (Scheme 3). Thus, despite being conceptually elegant and promising, this approach is significantly hampered by ^18^F^−^ solubility issues which will possibly be addressed in the future to establish this methodology as a practical alternative to the simpler and straightforward SiFA-leaving group method or IE methodology.

## 3. SiFA Lipophilicity and Hydrolytic Stability

**Figure 3:**
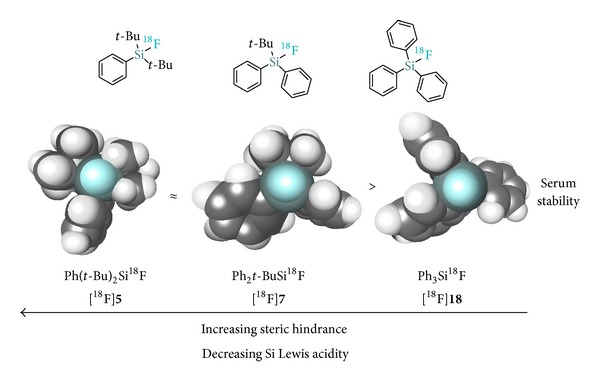
In vitro hydrolytic stability of [^18^F]fluorosilanes in human serum.

Stability investigations of a series of phenyl- and* tert*-butyl-bearing [^18^F]SiFAs ([^18^F]**5**, [^18^F]**7**, and [^18^F]**18**) early on established the importance of the* tert*-butyl substituents at the silicon atom in order to achieve sufficient in vivo stability for potential in vivo PET applications (Figure 3) [5]. Compound [^18^F]**18** displayed poor in vitro stability in human serum at 37.4°C (*t*_1/2_ = 5 min) while both [^18^F]**5** and [^18^F]**7** were found to be persistently stable under those conditions. However, only [^18^F]-*t*Bu_2_PhSiF ([^18^F]**5**) showed satisfactory in vivo stability as demonstrated by the limited ^18^F^−^ bone uptake observed upon injection into Sprague Dawley rats. The stability trend originates from steric hindrance in combination with the diminished silicon Lewis acidity in the presence of* tert*-butyl fragments. Unfortunately, this substitution pattern comes at the price of a significant increase in lipophilicity which, when chemically linked to biomolecules, may substantially impact metabolism and biodistribution, generating unspecific uptake and leading to poor PET imaging quality. This issue has been addressed by the development of lipophilicity-reducing auxiliaries which will be discussed in Section 4.

Further confirmation of the importance of sterically demanding SiFA substituents was provided by the detailed and systematic investigation on hydrolytic stability led by Höhne et al. (Table 1) [33].

**Scheme 4:**
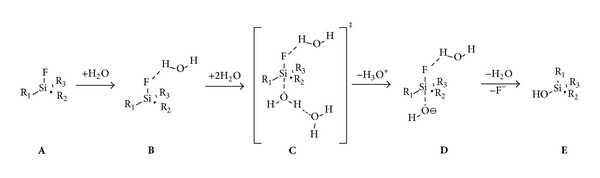
Mechanism for the hydrolysis of organofluorosilanes as suggested by Höhne et al.

The observed trends strongly correlate with the steric nature of the silicon substituents. In particular, the presence of bulky* tert*-butyl groups, combined with an aryl linker moiety, result in remarkable stability whereas smaller alkyl substituents progressively enhance the hydrolysis rate. Furthermore, the authors also provided a detailed hydrolysis mechanism (Scheme 4) as well as a theoretical model based on the difference in Si–F bond lengths (Δ_(Si−F)_) between the starting SiFA structures (**A**) and the DFT optimized intermediate structure (**D**) (where Δ_(Si−F)_ ≥ 0.19 Å corresponds to hydrolytically unstable SiFAs).

**Scheme 5:**
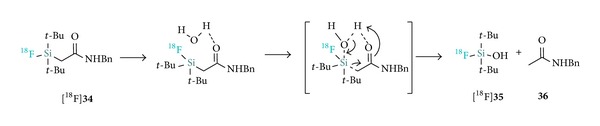
Suggested mechanism for the hydrolysis of [^18^F]SiFA *β*-acetamide [^18^F]**34**.

In a recent study, the group of Ametamey attempted the radiosynthesis of a *β*-acetamide [^18^F]SiFA ([^18^F]**34**) from the corresponding hydrosilane precursor but instead isolated di-*tert*-butyl-[^18^F]fluorosilanol ([^18^F]**35**) (Scheme 5) [36]. They suggested that this conversion proceeds with an analogous mechanism to the one encountered in the hydrolysis of *β*-ketosilanes following treatment with water [37]. This interestingly constitutes the first example of a SiFA hydrolytic stability issue involving the cleavage of the silicon-carbon bond.

## 4. [^**18**^F]SiFA Labeling of Peptides

The labelling of peptides for PET imaging has traditionally been achieved via multistep strategies involving ^18^F-S_*N*_2 reactions at carbon centers and ^18^F-labeled prosthetic groups. This strategy succeeded in generating multiple peptide-based PET probes for in vivo imaging [38–40] but it is inherently hampered by its technical complexity, harsh reaction conditions, and time-consuming HPLC purifications. Simplifying such procedures by means of mild and efficient radiolabeling approaches without HPLC purifications at one or all synthetic stages while maintaining sufficient SA represents an important challenge in ^18^F-PET radiochemistry. The [^18^F]SiFA method, as well as other promising emerging technologies such as the Al-^18^F approach [14–19], is particularly well suited to address those classical limitations.

**Scheme 6:**
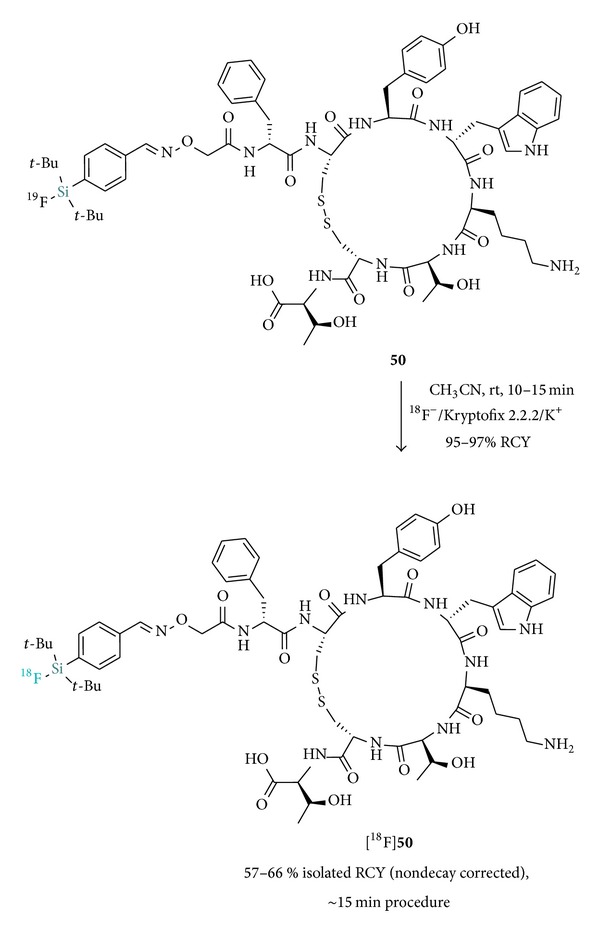
Radiosynthesis of [^18^F]SiFA-derivatized Tyr^3^-octreotate ([^18^F]**50**).

**Figure 4:**
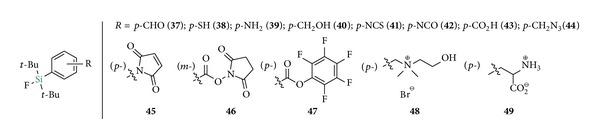
Structures of SiFA building blocks amenable to IE and peptide labeling.

Figure 4 presents various synthesized SiFA building blocks bearing reactive groups for peptide conjugation (for proteins and small molecules* vide infra*) [6, 32, 41–45]. The coupling of those SiFAs to peptides prior to the IE labeling would in theory allow for a direct and mild ^18^F-incorporation without subsequent HPLC purification. Indeed, this was early demonstrated by Schirrmacher et al. [5] with the direct radiosynthesis of [^18^F]SiFA-derivatized Tyr^3^-octreotate ([^18^F]**50**, Scheme 6). Despite the unprecedented mild conditions encountered and the high ^18^F-fluorination efficiency of 95–97% and 57–66% isolated RCYs (nondecay corrected), the approach suffered from low SAs (0.08–0.14 Ci *μ*mol^−1^).

**Scheme 7:**
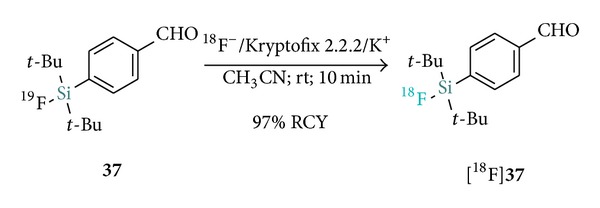
Radiosynthesis of [^18^F]-SiFA-*p*-CHO ([^18^F]**37**) for the labeling of aminooxy derivatized peptides.

Subsequently, a two-step procedure which consists of the near quantitative initial fluorination of the aldehyde [^18^F]**37** (Scheme 7) in high SAs (>5000 Ci/mmol), followed by a rapid C-18 SPE purification and subsequent room temperature conjugation to* N*-terminal amino-oxy functionalized Tyr^3^-octreotate, was reported [29] (Table 2 recapitulates selected examples of SiFA-peptide labeling). In the same study, the [^18^F]**37** synthon was also efficiently applied to the labeling of a cyclic RGD (Arg-Gly-Asp) and a PEG-conjugated bombesin (BBN) analogue (*cyclo*(fK(AO-N)RGD and BZH3, resp.).

**Figure 5:**
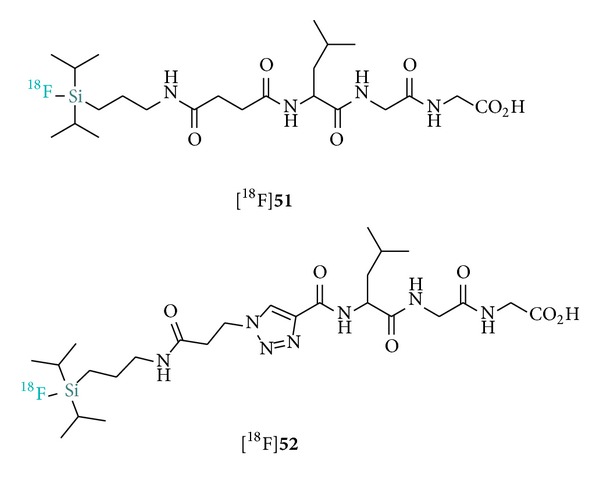
Hydrolytically unstable di-*i*Pr-SiFAs tripeptides reported by Balentova et al.

In parallel, important progress towards the direct fluorination of bioactive peptides from hydrosilanes and silanol precursors following the leaving group approach was made. The initial report by Mu et al. illustrates the methodology with the synthesis of two ^18^F-labeled tetrapeptides. The reactions proceeded at 65–90°C with moderate incorporation of ^18^F from either of the hydrosilane and the silanol (45% and 53%, resp.) [21]. The importance of the bulky* t*Bu_2_Ph-SiFA motif to guarantee hydrolytic stability was confirmed once more. Both an* i*Pr_2_Ph-SiFA bombesin analogue [33] and two alkyl-linked* i*Pr_2_-SiFA model tripeptides were shown to be unstable (pH 7.5, phosphate buffer) [46] (Figure 5). Following the leaving group approach, the development and first in vivo evaluation of a [^18^F]SiFA labeled bombesin analogue in PC3 xenografted nude mice were subsequently reported [9, 33] (Table 2, Entry 7). The authors reported low uptake in gastrin-releasing peptide receptor (GRP) positive tumor bearing mice and high unspecific binding along with prominent hepatobiliary excretion, despite sufficient potency (IC_50_ = 22.9 nM) based on comparison with previously characterized successfully radiolabeled BBN analogues. The observation of gradually increasing but overall low bone uptake suggested that di-*tert*-butyl aryl [^18^F]SiFA was sufficiently stable in vivo. Hence, the poor pharmacokinetic profile observed was reasonably ascribed to the overall high lipophilicity of the probe imparted by the SiFA moiety.

Wängler et al. reported the synthesis, HPLC-free purification, and in vivo evaluation of carbohydrate and carbohydrate/PEG derivatized [^18^F]SiFA-octreotate probes for imaging sst2-expressing tumors (AR42J xenografts; Table 2; Entries 3 and 4). [47]. This study, based on the previously successful use of hydrophilic linkers for enhanced tumor uptake and optimized excretion of PET/SPECT imaging peptides introduced by Schottelius and Antunes et al. [48–50], established the efficiency of peptide SiFA derivatives with lipophilicity-reducing auxiliaries as a potential strategy for optimized PET imaging. The in vivo investigation of the most promising PEG/glucose-linked derivative ([^18^F]SiFA-Asn(AcNH-*β*-Glc)-PEG-Tyr^3^-octrotate – IC_50_(sst2) = 3.3 ± 0.3 nM; Table 2, Entry 4) showed enhanced tumor uptake (7.7% ID/g at 60 min p.i.) compared to the initial negligibly accumulating [^18^F]-SiFA-Tyr^3^-octreotate (entry 1). This positive, yet still nonoptimal result was attributed to the improved hydrophilicity of the probe (log⁡ *P*_*ow*_ = 0.96 versus 1.59 for [^18^F]-SiFA-Tyr^3^-octreotate) and encouraged the introduction of hydrophilic auxiliaries as a promising lipophilicity counterbalancing strategy for SiFA-peptide probe development. This approach has since been translated into a general procedure aiming at the modular cartridge-based radiosynthesis of various [^18^F]SiFA peptides in conjunction with lipophilicity-reducing auxiliaries [51].

Two recent additional studies described further lipophilicity reducing auxiliaries for SiFA-peptides. Firstly, Amigues et al. introduced a PEG/ribose [^18^F]-SiFA-RGD probe ([^18^F]SiFA-RiboRGD; Table 2, Entry 8) as a silicon-based alternative with counterbalanced lipophilicity to the well-known [^18^F]Galacto-RGD [52, 53]. [^18^F]SiFA-RiboRGD was obtained from the corresponding hydrosilane in satisfactory yields and SA (Table 2) and the in vivo PET evaluation suggested that the tracer might be useful in the determination of *α*v*β*3 integrin expression as significant tumor uptake was reported.

**Scheme 8:**
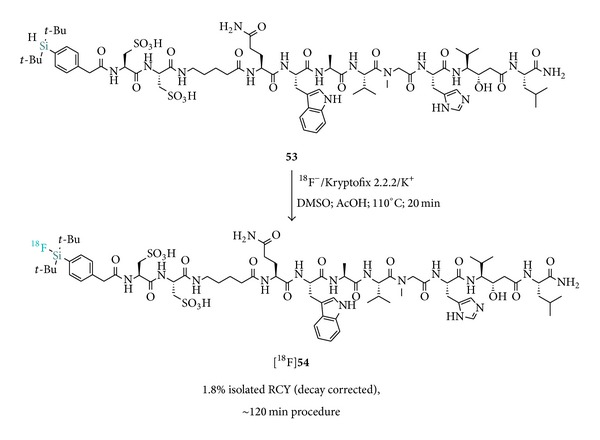
Radiosynthesis of l-cysteic acid-containing SiFA bombesin analogue [^18^F]**54**.

Secondly, the group of Ametamey introduced another lipophilicity reducing strategy towards the development of optimized [^18^F]SiFA bombesin analogues [36]. The synthesis of tartaric acid/l-cysteic acid-containing linked BBN derivatives allowed for a significant lipophilicity reduction (log* D*_7.4_ = 0.3 ± 0.1 for [^18^F]**54** versus 1.3 ± 0.1 for cysteic acid free peptide-entry 7, Table 2). The in vivo evaluation of the most potent derivative [^18^F]**54**, which was labeled in low overall RCY of 1.8% from the hydrosilane** 53**, demonstrated that the positive physicochemical alteration introduced by the hydrophilic auxiliary correlated with improved imaging properties (Scheme 8). Enhanced tumor accumulation and tumor-to-blood ratio were detected in PC-3 xenografted mice compared to the lipophilic [^18^F]SiFA-BBN probe.

## 5. [^**18**^F]SiFA Protein Labeling

The ^18^F-labeling of large biomolecules, such as proteins, antibodies, and more recently affibodies, has traditionally been accomplished by ^18^F-carbon prosthetic labeling agents such as [^18^F]fluorobenzaldehyde ([^18^F]FBA),* N*-(2-[4-([^18^F]fluorobenzamido)ethyl]maleimide ([^18^F]FBEM), and * N*-succinimidyl 4-[^18^F]fluorobenzoate ([^18^F]SFB) [54–57]. Notwithstanding successful conjugation of those prosthetic groups to various proteins, their conjugation normally requires multiple hours of technical manipulations from the initial ^18^F^−^ drying to the delivery of the labeled proteins. SiFA-IE, which proceeds rapidly and efficiently under mild conditions, offers much simplified procedures towards ^18^F-labeled proteins.

**Scheme 9:**
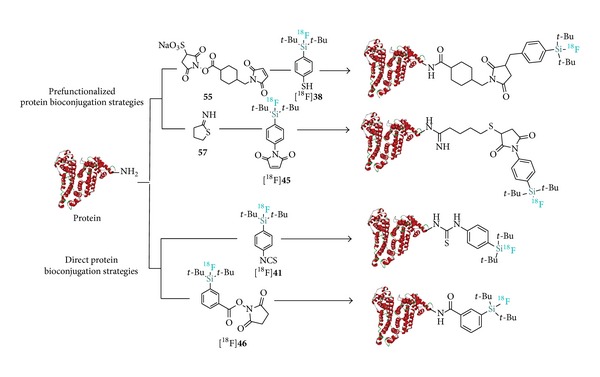
Strategies towards the synthesis of [^18^F]SiFA-labeled proteins by means of [^18^F]SiFA prosthetic groups.

Initial attempts to radiolabel active esters such as* N*-succinimidyl 3-(di-*tert*-butyl[^18^F]fluorosilyl)benzoate** 46** ([^18^F]SiFB) and the pentafluorophenyl ester** 47** for protein labeling failed even under IE conditions due to the propensity of those reactive moieties to hydrolyze under even slightly basic conditions. As an alternative approach, Iovkova et al. designed a prefunctionalization strategy involving protein derivatization with 2-iminithiolane (**57**) followed by the reaction with the SiFA maleimide [^18^F]**45** for the labeling of rat serum albumin (RSA) used for blood pool PET imaging (Scheme 9) [45]. The derivatization strategy was also applied with success to RSA labeling with [^18^F]SiFA-SH ([^18^F]**38**) [6]. Protein functionalization with sulfo-SMCC (**55**) followed by treatment with [^18^F]SiFA-SH obtained by IE allowed for the isolation of [^18^F]SiFA-RSA in overall 12% RCY within 20–30 minutes. An important improvement towards simplified labeling was reported by Rosa-Neto et al. with the first introduction of a direct labeling agent, [^18^F]SiFA-isothiocyanate ([^18^F]**41**) which obviates preceding protein derivatization [44]. Remarkably, and despite the high reactivity of the isothiocyanate fragment, the IE proceeded nearly in quantitative yields (95% RCY; rt; 10 min) and allowed for the efficient direct synthesis of various ^18^F-labeled model proteins (RSA, apotransferrin, and bovine IgG) in suitable SAs (2.7–4.5 Ci *μ*mol^−1^).

Subsequently, the decomposition of active esters such as [^18^F]SiFB ([^18^F]**46**) during radiolabeling due to the basicity of the reaction mixture (potassium oxalate/hydroxide) was resolved by addition of a suitable amount of oxalic acid in order to neutralize the base present during the labeling procedure [42]. This study showed the feasibility of the cartridge-based synthesis of [^18^F]SiFB and demonstrated the applicability of this labeling synthon for protein labeling. This new SiFA based approach is technically much less demanding than the radiosynthesis of the well-known* N*-succinimidyl 4-[^18^F]fluorobenzoate ([^18^F]SFB), providing a simple access to ^18^F-labeled proteins. This has led to the report of a standardized protocol for protein labeling via SiFB [58]. A straightforward labeling protocol has also been reported for protein labeling with [^18^F]SiFA-SH ([^18^F]**38**) [59].

**Scheme 10:**
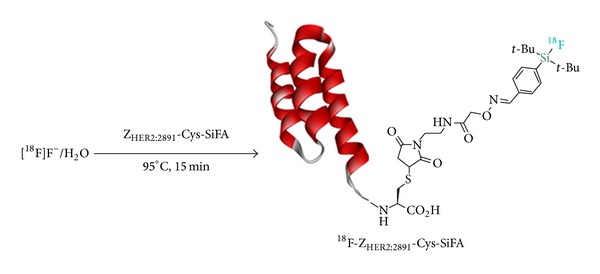
Aqueous IE radiosynthesis of [^18^F]-Z_HER2:2891_-Cys-SiFA by Glaser et al.

The scope of SiFA-IE has recently been extended to the labeling of affibodies. Glaser et al. reported the efficient synthesis of a cysteine modified human epidermal growth factor receptor (HER2)-targeted affibody, [^18^F]Z_HER2:2891_ -Cys-SiFA (Scheme 10) [60]. This study demonstrated the convenience and selectivity of the IE at a silicon-atom with the efficient aqueous radiolabeling of [^19^F]-Z_HER2:2891_-Cys-SiFA precursor from [^18^F]F^−^/[^18^O]H_2_O. Similar aqueous procedures had previously been described for the synthesis of a small SiFA-octreotate derivative (Scheme 7, [^18^F]**50**) by Schirrmacher et al. [5]; however, direct aqueous labeling of large biomolecules such as affibodies (58 amino acids) is remarkable. Comparison with [^18^F]benzaldehyde ([^18^F]-FBA) and [^18^F]Al-F/NOTA protocols conclusively demonstrated the efficiency of the SiFA-IE technique in terms of synthesis (RCYs, purity, and SA) despite an observed inferior in vivo profile, mainly attributed to hydrolysis leading to ^18^F bone uptake.

## 6. Towards a Kit Formulation for SiFA-IE

**Figure 6:**
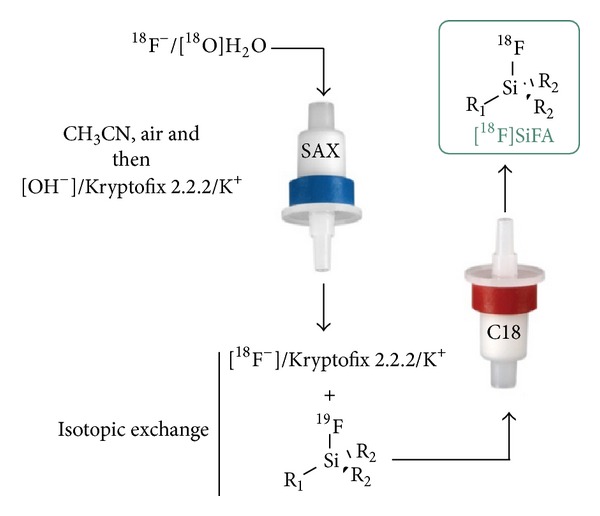
Combination of SiFA-IE strategy with the “Munich” ^18^F drying method. The combination of the “Munich method” and the simple cartridge purification achievable by IE allows for a simple kit production procedure.

Recently, a new drying method known as the “Munich method” has been introduced by Wessmann et al. which significantly simplified ^18^F radiochemistry compared to the more classical and time-consuming azeotropic drying of ^18^F^−^ [61]. The technique consists of the elution of dry ^18^F^−^ from an anion exchange cartridge (SAX) with lyophilized Kryptofix 2.2.2./potassium hydroxide complex dissolved in anhydrous acetonitrile (Figure 6).

This procedure is fast (3–5 min) and fully devoid of azeotropic drying and is easily implemented into an automated setup. The recent implementation of the “Munich method” alongside the SiFA-IE labeling approach for peptide and protein labeling [43, 46, 58, 59] offers unique and unequalled simplicity, where, starting from commercially available [^18^F]F^−^/[^18^O]H_2_O, it is possible to deliver [^18^F]SiFA radiopharmaceuticals using only room temperature transformations and facile cartridge-based manipulations. Following this approach, the ^18^F-labeling of complex unprotected biomolecules becomes almost as easy as using a ^99m^Tc-kit.

## 7. Small Molecules

It has previously been shown that, in the absence of suitable auxiliaries, the intrinsic lipophilicity introduced by the SiFA moiety often results in significant alteration of the overall physicochemical properties and in vivo biodistribution of the bioactive compound to which they are bound. This is especially true for ligands with low molecular weight. Nevertheless, certain groups have studied ^18^F-radiolabeled silicon-based small ligands for PET imaging and, in some cases, obtained preliminary useful in vivo PET data.

An initial study by Bohn et al. and a follow-up investigation by Joyard et al. demonstrated the synthesis, radiolabeling, and in vivo evaluation of silicon-based analogues of [^18^F]FMISO, an established tracer for detection of hypoxia [62, 63]. In spite of the well-known steric requirements of the silicon atom, the authors described a series of alkyl substituted [^18^F]SiFA-FMISO analogues which resulted in insufficient hydrolytic stability both in vitro and in vivo (Table 3; Entries 1 and 2). Accordingly, the dimethyl [^18^F]SiFA MISO compound (*t*_1/2_ < 5 min) only showed poor tumor uptake in mice while radioactivity accumulation occurred rapidly and significantly in bones due to the in vivo liberation of ^18^F^−^. The more stable dinaphthyl derivative (*t*_1/2_ = 125 min) (Entry 4, Table 3) was retained in pulmonary capillaries due to its high lipophilicity (cLog P = 6.47). After evaluating other unstable derivatives, the authors described the synthesis and evaluation of a promising* t*Bu_2_Ph-based [^18^F]SiFA tracer (Entry 7, Table 3) which was sufficiently stable for in vivo PET evaluations in rat. Upon injection, the tracer was shown to be heterogeneously distributed in healthy rats but unfortunately no evaluation in animals bearing a hypoxic tumor was reported.

**Figure 7:**
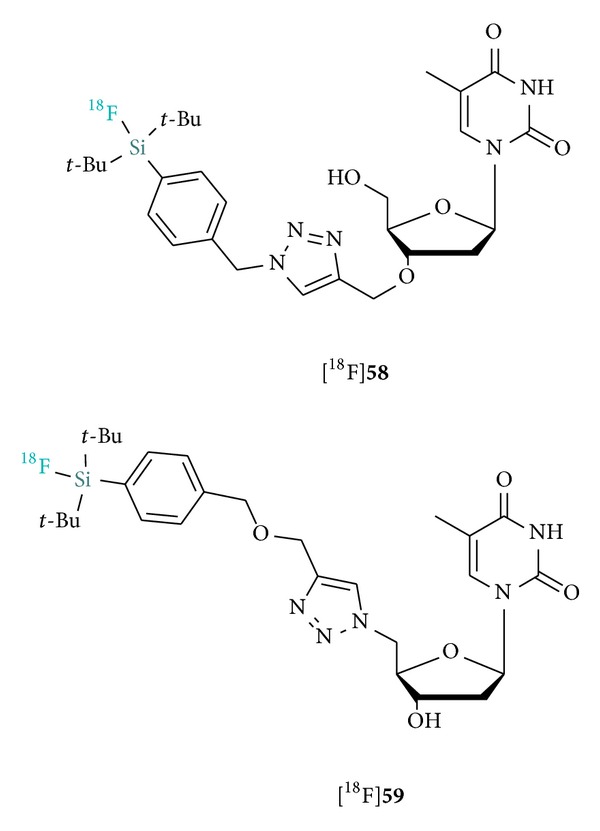
Structures of ^18^F-labeled thymidine probes.

Recently, Schulz et al. reported a protocol for the efficient radiolabeling of nucleosides and nucleotides derivatized with the SiFA building block. The labeled silylated thymidines [^18^F]**58** and [^18^F]**59 **were obtained in high SA (10 Ci *μ*mol^−1^) from the corresponding hydrosilanes in 43% and 34% RCYs, respectively (Figure 7) [64]. Despite the potential application of those SiFA tracers as [^18^F]FLT surrogates, no in vivo data is currently available. The described procedure was also applied to the ^18^F-radiolabeling of di- and oligonucleotide probes.

**Figure 8:**
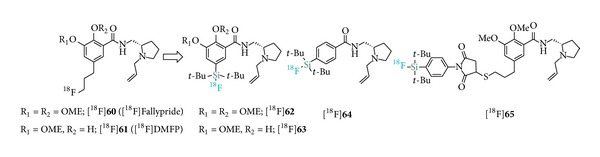
Structures of [^18^F]SiFA D_2_-receptor ligands.

In a thorough study, silicon-based D_2_-receptor ligands with structures analogous to [^18^F]fallypride ([^18^F]**60**) and [^18^F]desmethoxyfallypride ([^18^F]**61**) were reported (Figure 8) [65]. Derivatization with SiFA resulted in 44–650 times decreased affinities towards the D_2_-receptor compared to fallypride (*K*_*i*_ = 0.0965 ± 0.0153 nM), yet remaining in the low nanomolar range. Upon optimization, the IE strategy delivered tracers [^18^F]**62**, [^18^F]**63**, and [^18^F]**64** in 54–61% RCYs and all three tracers could be purified by just using SPE techniques. The measured SAs were in the range of 1.1–2.4 Ci *μ*mol^−1^. The most potent derivative, [^18^F]**65 **(*K*_*i*_ = 4.21 ± 0.41 nM), was labeled in only the modest RCY while stability issues prevented its purification following the solid-phase method. In vivo PET data were not reported.

**Figure 9:**
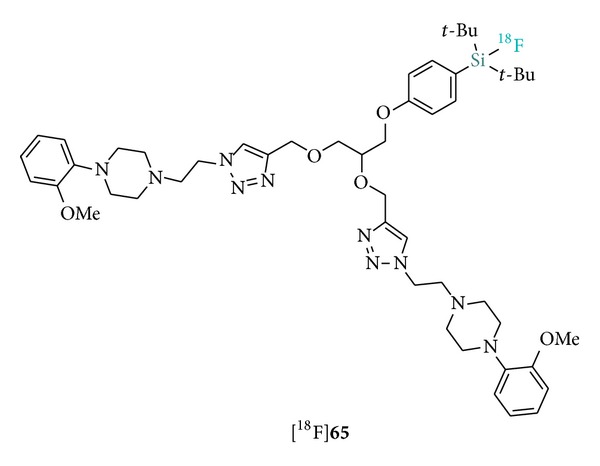
Structure of the dimeric 5-HT_1A_ radioligand [^18^F]BMPPSiF.

The most recent contribution from Hazari et al. describes the design and evaluation of a highly potent and selective 5-HT_1A_ homodimeric SiFA-dipropargyl glycerol derivatized radioligand aimed at PET imaging of dimeric serotonin receptors (Figure 9; [^18^F]**65**) [66]. This multimeric approach is supported by the development of bivalent 5-HT ligands based on recent evidence suggesting that some 5-HT receptors exist as dimers/oligomers [67]. The tracer, [^18^F]BMPPSiF, was obtained following the leaving group approach from the corresponding hydrosilane. The synthesis of the precursor was achieved via double azide-alkyne Huisgen cycloaddition with two azidoethyl (2-methoxyphenyl)piperazine fragments. Subsequent ^18^F-radiofluorination occurred in 52 ± 10.5% RCY upon heating to yield [^18^F]BMPPSiF with a SA of 13 Ci *μ*mol^−1^. Brain PET imaging in rats showed high uptake in 5-HT_1A_ receptor rich regions. As expected, significant reduction of the uptake in the hippocampus was detected in serotonin-depleted rat models. Blocking studies did not reveal significant decrease in uptake. Notably, this report constitutes the first example of a SiFA-small ligand with positive PET imaging data. Interestingly, it also suggests that when applicable, [^18^F]SiFA-based multimeric derivatization may help compensate the overall influence on physicochemical parameters of the SiFA moiety on small ligands.

## 8. SiFA: A Critical Assessment

From the very first appearance of SiFA compounds in 2006 and 2008 the groups of Ametamey and Schirrmacher/Wängler/Jurkschat have put extensive efforts into the structural optimization of the SiFA building blocks. The main drawback of this labeling technique irrespective of the actual labeling methodology (IE or leaving group approach) is the inherently extremely high lipophilicity hampering in vivo application in general. The compounds of the first generation when injected into animals were almost exclusively metabolized by the hepatobiliary system which lead to a high liver uptake and almost zero uptake in the target tissue. Both groups have approached this problem by introducing hydrophilic components into the SiFA tagged molecules to compensate for the high lipophilicity. However, this strategy is only useful for larger biomolecules such as peptides and proteins which tolerate an extensive structural modification. It could be convincingly demonstrated by Niedermoser et al. recently that highly hydrophilic SiFA derivatized somatostatin analogues can be labeled in a one-step reaction via IE in high RCYs and SAs of 1200–1700 Ci/mmol [68]. High IC_50_ values of the SiFA-peptides in the low nanomolar range and a very high tumor uptake of >15% in a AR42J nude mice tumor model showed that the lipophilicity problem has been successfully solved, paving the way for a human clinical application in the near future. The most recently published paper by Lindner at al. demonstrated that SiFA tagged RGD peptides can serve as tumor imaging agents in a mouse U87MG tumor model if hydrophilic auxiliaries are added in combination with the SiFA labeling moiety [69]. A tumor uptake of 5.3% ID/g was observed, clearly delineating the tumor from other tissues. Unfortunately smaller molecules lend themselves less towards a SiFA labeling because of the difficulty of compensating for the SiFA lipophilicity. A small molecule such as a typical receptor ligand for brain imaging does not accept considerable structural modifications to adjust the SiFA lipophilicity without seriously compromising its binding properties to the target receptor. It is therefore unlikely that the SiFA labeling technique will grow into a staple for labeling molecules of small molecular weight. It is also true that all compounds reported so far have been only used in animal experiments. The SiFA methodology still has to prove its usefulness in a human clinical setting. This however requires extensive efforts and financial commitments from the academic research groups and it is hoped that the industry, which already showed interest in this labeling technique, will help transitioning this promising labeling technique to the clinic.

## 9. Conclusion

The SiFA methodology has grown over the years from a niche methodology to a broadly applied labeling strategy towards innovative ^18^F-labeled radiopharmaceuticals for PET. SiFA radiolabeling procedures have been methodically studied and can be easily performed using either the SiFA leaving group approach or the SiFA-IE methodology. Moreover, those approaches are now well-established for a great variety of structurally distinct high affinity probes such as peptides, proteins, affibodies, and even small ligands. The practical simplicity and mild reaction conditions of the SiFA-IE strategy in particular represents a unique advantage in ^18^F-labeling which, when applied in synergy with the recently developed Munich drying method, helps meeting the requirements for a true kit-like ^18^F-labeling procedure.

## Figures and Tables

**Table 1:** Hydrolytic half-lives (*t*_1/2_) of selected organofluorosilane building blocks.

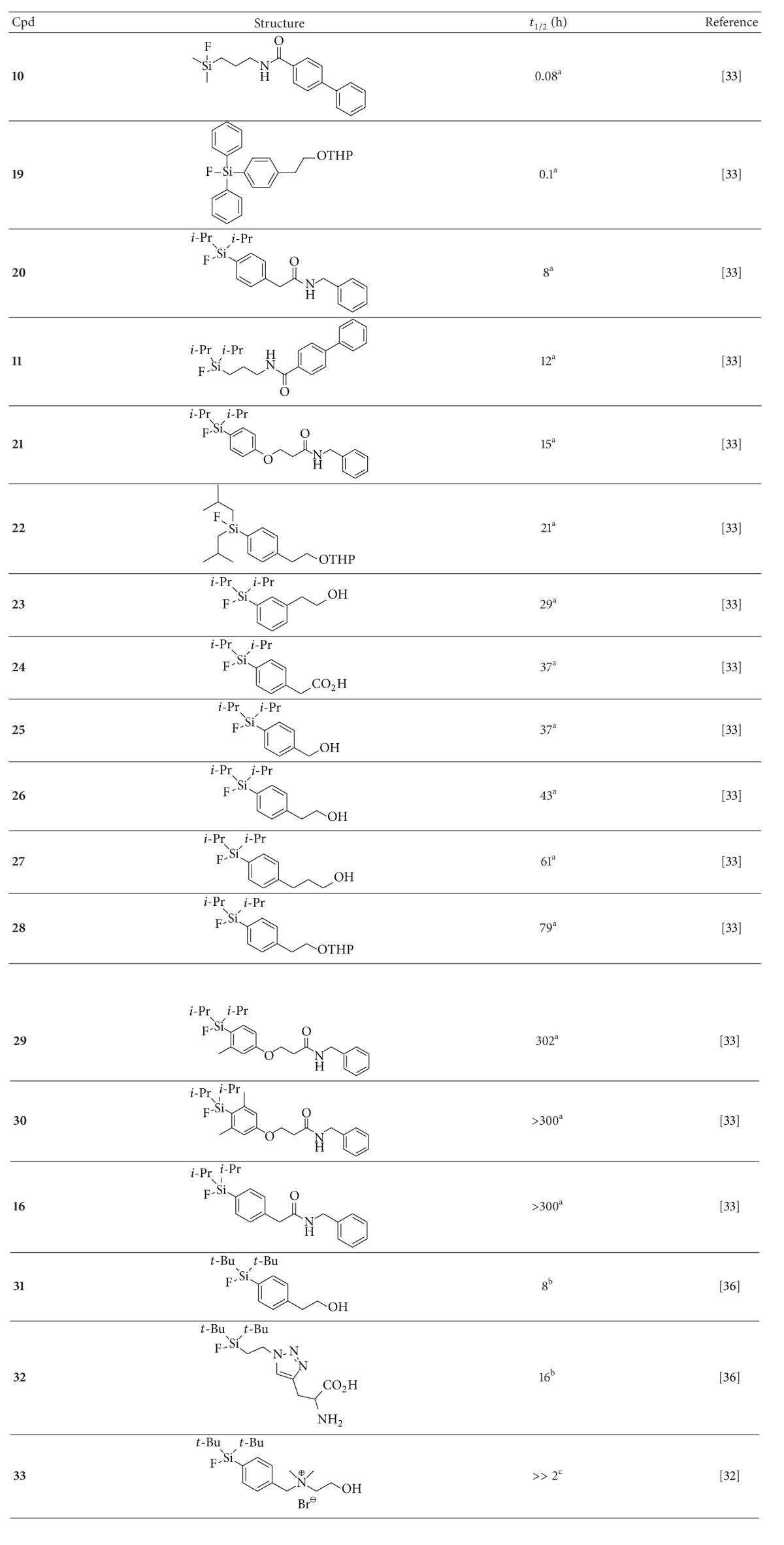

^a^Hydrolytic stability determination from nonradioactive compounds in MeCN/aqueous buffer (2 : 1; pH 7) at room temperature. ^b^Hydrolytic stability determination from ^18^F-labeled compounds in EtOH/aqueous buffer at room temperature. ^c^95% intact after 2 h of incubation; hydrolytic stability determination from ^18^F-labeled compounds at pH 7.4.

**Table 2:** Structure of selected [^18^F]-silicon-based derivatives attached to different peptide ligands and their appended linkers and lipophilicity-reducing auxiliaries.

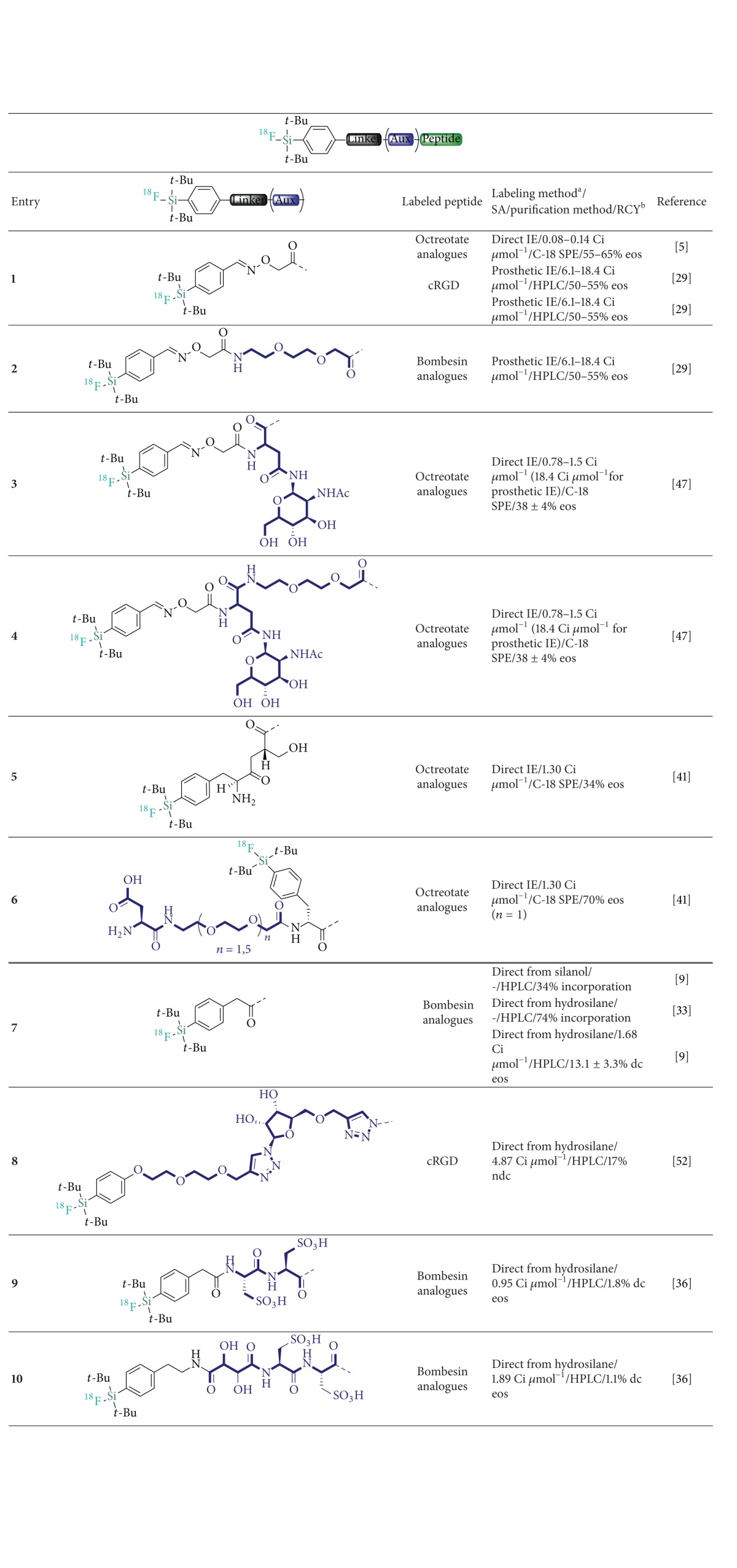

^a^Via isotopic exchange (IE) either direct or in two steps or via the leaving group approach from the specified precursor. ^b^The RCYs are reported as isolated end of synthesis (eos) yields either decay correct (dc) or not (ndc); in the absence of available RCYs at eos, incorporation RCYs are reported.

**Table 3:** Structures of ^18^F-silicon-based nitroimidazoles for PET hypoxia imaging.

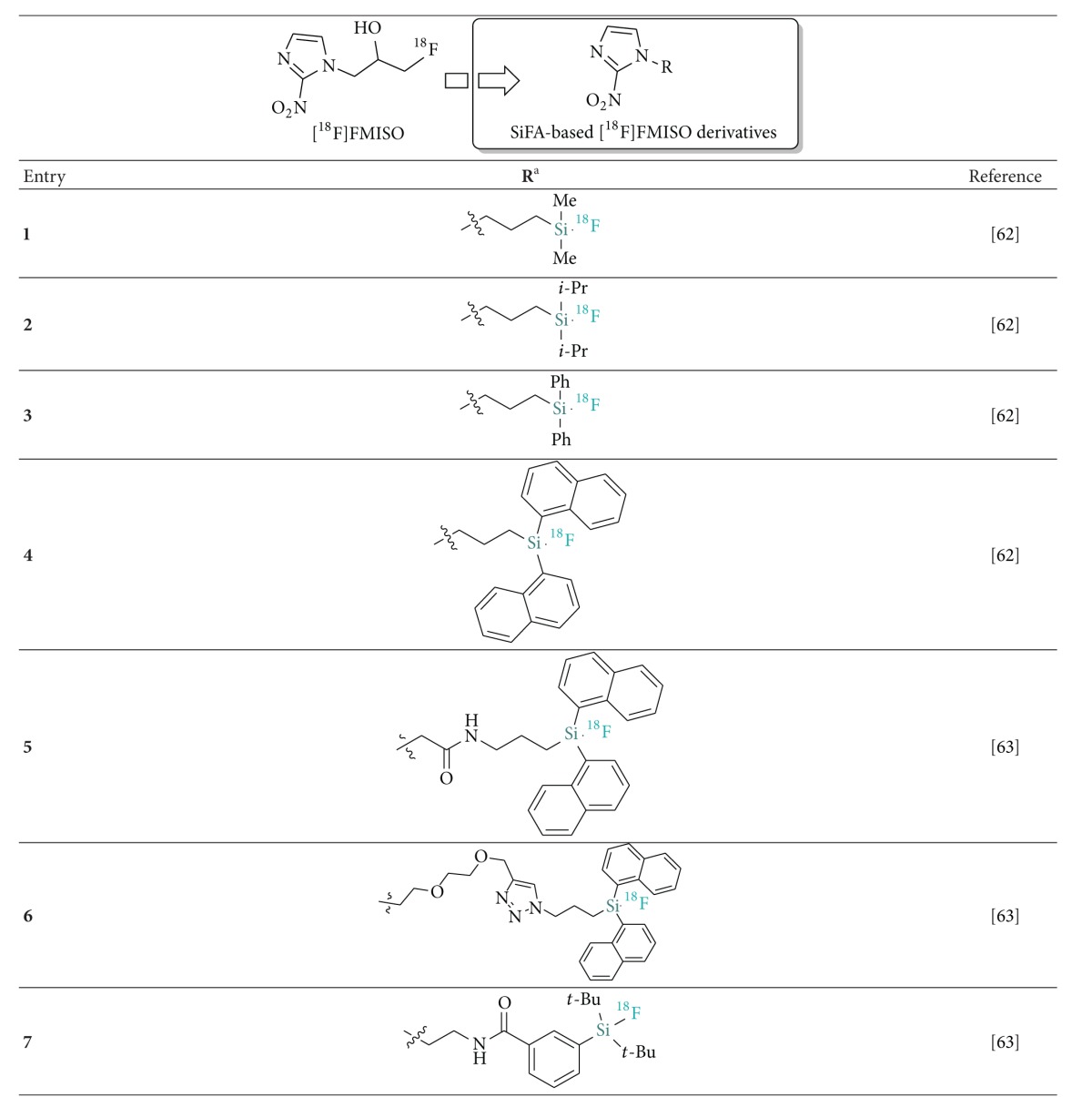

^a^Tracers were obtained via the SiFA leaving group approach from the corresponding silyl ethers.

## Abbreviations

PET: Positron emission tomography
SiFA: Silicon-fluoride-acceptor
IE: Isotopic exchange
HPLC: High-performance liquid chromatography
D_2_ receptor: Dopamine receptor subtype D_2_
RCY: Radiochemical yield
SPECT: Single-photon emission computed tomography
GMP: Good Manufacturing Practice
Nca: No-carrier-added
DFT: Density functional theory
BDE: Bond dissociation energy
SPE: Solid phase extraction
NALG: Nucleophile assisting leaving groups
S_*N*_2: Nucleophilic substitution bimolecular
BBN: Bombesin
GRPR: Gastrin-releasing peptide receptor
PEG: Polyethylene glycol
SA: Specific radioactivity
RSA: Rat serum albumin
SAX: Anion exchange cartridge
5-HT_1A_: Serotonin receptor.
